# Mutagenicity testing with transgenic mice. Part I: Comparison with the mouse bone marrow micronucleus test

**DOI:** 10.1186/1477-3163-4-3

**Published:** 2005-01-17

**Authors:** U Wahnschaffe, A Bitsch, J Kielhorn, I Mangelsdorf

**Affiliations:** 1 Fraunhofer Institute of Toxicology and Experimental Medicine ITEM, Department of Chemical Risk Assessment, Nikolai-Fuchs-Str. 1, 30625 Hannover, Germany

## Abstract

As part of a larger literature study on transgenic animals in mutagenicity testing, test results from the transgenic mutagenicity assays (*lacI *model; commercially available as the Big Blue^® ^mouse, and the *lacZ *model; commercially available as the Muta™Mouse), were compared with the results on the same substances in the more traditional mouse bone marrow micronucleus test. 39 substances were found which had been tested in the micronucleus assay and in the above transgenic mouse systems. Although, the transgenic animal mutation assay is not directly comparable with the micronucleus test, because different genetic endpoints are examined: chromosome aberration versus gene mutation, the results for the majority of substances were in agreement. Both test systems, the transgenic mouse assay and the mouse bone marrow micronucleus test, have advantages and they complement each other. However, the transgenic animal assay has some distinct advantages over the micronucleus test: it is not restricted to one target organ and detects systemic as well as local mutagenic effects.

## Background

This and the following presentation are part of a project for the International Programme on Chemical Safety (IPCS) evaluating the possible use of transgenic animal mutagenicity assays in chemical toxicity testing and mechanistic research. It was decided to compare the results obtained from those transgenic mutagenicity test systems where considerable data was available, with other in vivo genotoxicity tests: in the first article (Part 1) with the mouse bone marrow micronucleus test and in the second (Part II) with the mouse spot test.

The mouse bone marrow micronucleus test is one of several available *in vivo *mammalian test system for the detection of chromosomal aberrations [1-5]. A documentation of the test procedure and evaluation of results is given in the OECD guideline 474 [6]. This test is routinely used with a widespread acceptance in industry and authorities.

Mutagenicity assays using transgenic animals have been developed in particular the *lacI *model [7] (commercially available as the Big Blue^® ^mouse), and the *lacZ *model [8] (commercially available as the Muta™Mouse). In this article, available data on the results of mouse bone marrow micronucleus test were compared with results from these two transgenic mouse assays for 39 substances. The advantages and disadvantages of the test systems are discussed. Recently, further transgenic rodent mutation assays have been developed, however, the data base is not sufficient for comparison with other test systems.

### The mouse bone marrow micronucleus test: principles and procedure

Micronuclei are chromatin-containing bodies in the cytoplasm arising from acentric chromosome fragments or from whole chromosomes that were not incorporated in the daughter nuclei during the last stages of mitosis. Chromosome fragments are associated with the clastogenic (chromosome breakage) activity of the test substance whereas the presence of a whole chromosome is indicative of an adverse effect of the test substance on the mitotic spindle apparatus (aneugenic effects). The difference in size of the micronucleus is therefore an indicator for clastogenicity (small micronucleus) or aneugenicity (large micronucleus). However, the size of the micronucleus is an inaccurate measure. Micronuclei can be distinguished by further criteria, for example by identification of the presence of a kinetochore or centromeric DNA, indicating aneugenic activity. Overall, an increase in micronuclei is an indirect measure of induced structural or numerical chromosome aberrations [1,2].

In the micronucleus test according to the OECD guideline 474, erythroblasts in the bone marrow of mice (or rats) are used as target cells. When a bone marrow erythroblast develops into a polychromatic erythrocyte, the main nucleus is extruded. Any micronucleus that has been formed may remain in the otherwise anucleated cytoplasm and can easily been detected. An increase in the frequency of micronucleated polychromatic erythrocytes in treated animals is an indication of induced chromosome damage [6].

In the last three decades, toxicologists have often used the mouse bone marrow micronucleus test because 1) it is part of the regulatory toxicology in the admission procedure for chemicals and drugs and 2) it has advantages in speed, simplicity, and cost effectiveness in comparison to other *in vivo *systems for testing chromosomal aberrations (e.g. the cytogenetic test).

### Transgenic mouse models

Transgenic mutation test systems contain a foreign gene construct having two essential parts: the transgene containing the *target gene *that serves as target for mutations, and a *shuttle vector *for recovering the target gene DNA from the tissue of the transgenic animal. The transgene is constructed using recombinant DNA technologies.

#### *LacI *transgenic mouse model (Big Blue^® ^mouse)

The proprietary mouse of the Big Blue^® ^mutagenesis assay system contains about 30–40 copies of a lambda LIZα shuttle phage vector integrated into its genome at a single locus on chromosome 4. The target site for mutagenesis is the *lacI *gene [7].

The test compound is administered to the mouse either as a single or repeated dose. After the post treatment period for manifestation of the DNA lesions, the tissue of interest is isolated and the DNA extracted. A proprietary lambda DNA packaging extract automatically excises the lambda vector target and packages it into a lambda phage head and the phage is transfected to bacteria. These bacteria are plated on agar indicator plates containing the chromogenic substance (X-gal). The phage transfected bacteria with mutations in the *lacI *gene form blue plaques, whereas bacteria with a nonmutated *lacI *form colourless plaques. The ratio between blue and white plaques is a measure of the mutagenicity. [7,9].

#### *LacZ *transgenic mouse model (Muta™Mouse)

The lambda-gt10-*lacZ *shuttle vector for the *lacZ *mouse model contains the entire lacZ target gene [8]. The commercially available *lacZ *mouse model contains about 80 copies of the shuttle vector at chromosome 3.

As above, the test compound is administered to the mouse, the genomic DNA is isolated from the tissue of interest, and the lambda genomes are excised by and packed with a bacteriophage packaging extract. The resulting phage particles are then plated on a *lacZ*^- ^*E. coli *strain (for transfection) in the presence of the chromogenic substance (X-gal) as indicator. The plaques containing an intact *lacZ *are β-galactosidase active and are blue, whereas plaques containing mutated *lacZ *will be white/colourless. In this original model the ratio between colourless and blue plaques is a measure of the mutagenicity. Due to optical difficulties in the evaluation of plaques, this system has been improved using a selection assay *in lieu *of colour screening whereby only mutant particles form plaques [10]. The number of plaques under non-selective conditions is a measure for the total number of phage-transfected bacteria (intact **or **mutant lacZ gene). The ratio between the number of plaques produced under selective conditions versus the number of plaques under non-selective conditions is a measure of the mutagenicity [9,10].

## Methods

Data presented in this documentation are the results of an extensive literature research. Concerning data on transgenic mouse assays only primary literature was used. Data on the mouse bone marrow micronucleus assay were extracted from reliable reviews on this item or from primary literature. For all other data informations from secondary literature or data compilation bases were used.

## Results and Discussion

### Comparison of data from the mouse bone marrow micronucleus test and transgenic mouse test

The authors are aware that a comparison of the transgenic mouse assays with the mouse bone marrow micronucleus test is limited by the fact that different genotoxic endpoints are studied in these two systems. In transgenic mouse assays, point mutations and small insertions and deletions are detected whereas in the mouse bone marrow assay, chromosome breakage leading to light microscopically visible micronuclei resulting from chromosome fragment or micronuclei originated from whole chromosomes are investigated. However, both point mutations and micronuclei may be induced by a single agent, so some overlap of results is to be expected.

From the literature, 39 substances were identified with data on the mouse bone marrow micronucleus test **and **the Muta™mouse assay (n = 29) or the Big Blue^® ^mouse assay (n = 21) or both transgenic mutation assays (n = 11); see Additional file 1 for references. Agreement between the Muta™mouse and the micronucleus test was seen with 18 out of 29 substances, no agreement with 8 substances and in 3 cases the comparison is inconclusive because of questionable results in the micronucleus assay. With the Big Blue^® ^mouse assay, the results obtained with 14 out of 21 substances agreed with results in the mouse micronucleus test, but 6 showed no agreement and another was inconclusive.

Most substances included in this comparison are also carcinogenic in long-term assays on mice. No data on carcinogenicity in mice are available for 4-acetylaminofluorene and negative results were obtained with bromomethane and 2,6-diaminotoluene.

### Carcinogenic substances with positive results in the bone marrow micronucleus and transgenic gene mutation assays

#### Studies with Muta™mouse

The following 17 substances were gene mutagenic in at least one of the examined organs in the Muta™mouse assay, induced micronuclei in mouse bone marrow and showed positive results in carcinogenicity studies on mice: 2-acetylaminofluorene, 4-aminobiphenyl, benzo(a)pyrene, 1,3-butadiene, chlorambucil, cyclophosphamide, 7,12-dimethylbenz(a)anthracene, ethylmethanesulfonate, N-ethyl-N-nitrosourea, methylmethane-sulfonate, N-methyl-N'-nitro-N-nitrosoguanidine, N-methyl-N-nitrosourea, 4-nitroquinoline-1-oxide, N-nitrosodimethylamine, procarbazine, quinoline, and urethane. Further studies on chromosome aberration *in vitro *and *in vivo *(see Additional file 1) supported the results in the micronucleus test, except data on 4-aminobiphenyl (negative micronucleus test in rats) and quinoline (see below).

Of the substances tested for mutagenic activity in bone marrow in Muta™mice, 14 gave positive results, only 1,3-butadiene, methylmethanesulfonate, and quinoline were negative, indicating that other organs are more sensitive. 1,3-Butadiene is clearly clastogenic in other studies on chromosome aberration in mice and induced gene mutation in the bone marrow of Big Blue^® ^mice (see Additional file 1). Quinoline, however, gave inconclusive results in other *in vivo *studies on clastogenic effects in bone marrow of rats and mice.

#### Studies with Big Blue^® ^mouse

12 substances showed gene mutagenic effects in at least one of the examined organs in the Big Blue^® ^mouse assay, chromosome mutagenic effects in the mouse bone marrow micronucleus assay and induced carcinogenic effects in long-term assays on mice,: 2-acetylaminofluorene, aflatoxin B1, benzene, benzo(a)pyrene, 1,3-butadiene, cyclophosphamide, 7,12-dimethylbenz(a)anthracene, ethylene oxide, N-ethyl-N-nitrosourea, N-methyl-N-nitrosourea, N-nitrosodimethylamine, urethane. In the Big Blue^® ^mouse assay the target organ bone marrow revealed also increased mutation frequencies induced by benzene, 1,3-butadiene, and 7,12-dimethylbenz(a)anthracene. However, negative results in bone marrow were obtained with cyclophosphamide, ethylene oxide, and N-nitrosodimethylamine, indicating that other target organs are more sensitive in the Big Blue^® ^mouse assay. All of these 12 substances induced also chromosome aberrations in majority of further *in vitro *and *in vivo *studies.

### Substances with carcinogenic effects in mice but no agreement between the transgenic mouse assay and the mouse bone marrow assay

#### Acrylamide

The inconclusive result in the bone marrow micronucleus test [4] is contradictory to the Muta™mouse assay [21-23] which shows mutagenic activity in the target organ bone marrow but also contradictory to other *in vivo *tests on the endpoint chromosome aberration including a cytogenetic test on mice [4,19,21]. Micronuclei were detected in spleen and testis of mice [4]. Overall, using other experimental design the mouse bone marrow micronucleus assay might give clearly positive results.

#### 2-Amino-3-methylimidazo(4,5-f)quinoline (IQ)

IQ is mutagenic in the liver of the Muta™mouse [34] but negative results were obtained in the mouse bone marrow micronucleus test [2,35]. This negative result is supported by a negative cytogenetic assay on mice. Inconclusive results were obtained in *in vitro *studies on chromosome aberrations but IQ induced micronuclei in rats (see Additional file 1). This discrepancy might be due to the possibility that a) the liver but not the bone marrow of mice is target organ (the liver but not the blood is target organ in carcinogenicity [33]) or b) IQ is less clastogenic than gene mutagenic in mice.

#### ortho-Anisidine

The target organ of carcinogenesis in mice (and humans) is the bladder [36]. In the Big Blue^® ^assay on mice mutagenic activity was detected in the bladder but not in the liver [37]. As expected, the mouse bone marrow micronucleus test [4,36] gave negative results (also in rats; see Additional file 1), because the bone marrow is presumably not a target organ of mutagenicity.

#### Asbestos crocidolite

Local carcinogenic effects were observed in carcinogenicity studies, the lung is the target organ after inhalation [38-40]. Mutagenic activity was detected in the lung of Big Blue^® ^mice after inhalation [41]. As expected, clearly no systemic effects in the bone marrow could be observed in the mouse micronucleus test [4].

#### 2,4-Diaminotoluene

The main target organ in carcinogenicity is the liver (also in rats) [81]. Positive results were reported in two Big Blue^® ^mouse assays examining the liver [82-84]. The mouse micronucleus test gave negative results [4], however, systemic effects in the bone marrow are not expected from carcinogenicity studies. Results of other studies on chromosome aberration *in vivo *are inconclusive (see Additional file 1).

#### Hydrazine

This substance induced no mutagenic effects in lung, liver, or bone marrow of the Muta™mouse which were target organs in mouse carcinogenicity studies [119,120]. The mouse bone marrow micronucleus test gave positive results after repeated application [4,119]. However, single exposure was used in the Muta™mouse assay [120]. Studies on other *in vivo *genotoxicity endpoints have shown almost negative results after single exposure but genotoxic activity after repeated application, for example in the mouse bone marrow micronucleus assay [4]. Positive results might be expected in the Muta™mouse assay using another experimental design since other *in vivo *as well as *in vitro *test systems revealed gene mutagenic effects.

#### Methyl methanesulfonate

Only weak mutagenic effects in the liver but no effects in bone marrow were observed in the Muta™mouse [18,124] and negative results in the Big Blue^® ^mouse [111-113,126]. In the mouse bone marrow micronucleus test this carcinogenic substance induced chromosome aberration [1,127]; other *in vitro *and *in vivo *assays clearly supported the clastogenic activity [121,122]. There is evidence that the chromosome mutagenic activity is detectable at much lower doses than the gene mutagenic activity. Tinwell et al. (1998) [18] have shown on Muta™mice a weak gene mutagenic effect in the liver but no effect in the bone marrow. The same dose induced in these animals a significant increase in bone marrow micronuclei indicating clear clastogenic activity. Overall, methyl methanesulfonate is more clastogenic than gene mutagenic.

#### Mitomycin C

A very similar situation is given with mitomycin C. No mutagenic activity was observed in the Muta™mouse assay in liver and bone marrow after single injection but the same dose induced chromosome aberrations in the bone marrow of the same mice [142]. The mouse bone marrow micronucleus test [1,47] and all *in vivo *and *in vitro *assays on the endpoint chromosome aberration revealed clearly positive results [139-141].

#### N-Nitrosodiethylamine

The main target organ in systemic carcinogenesis is the liver [146-148]. As expected, mutagenic effects were detected in the liver of the Muta™mouse, but none in the bone marrow [104,106,149,150]. The same mice showed also no micronuclei at these dose levels [104]. Consequently, the mouse bone marrow micronucleus test gave negative results indicating that this is not a target organ of genotoxicity. However, there is also evidence that this substance shows more gene mutagenic activity than clastogenic activity because other *in vivo *studies gave no clear indication for chromosome aberration (see Additional file 1).

#### N-Nitrosodi-N-propylamine

There is evidence that this substance shows more gene than chromosome mutagenic activity. Beside local carcinogenic effects in carcinogenicity studies on mice and rats systemic effects were located in the liver (mouse and rats) and in bone marrow (rat) [160-163]. Several target organs were detected in the Muta™mouse assay including liver and bone marrow [164]. But no chromosome mutagenic activity was recorded in the mouse bone marrow micronucleus test [4]. Further *in vivo *data on this endpoint are not available (see Additional file 1).

#### Phenobarbital

Liver tumours were detected in carcinogenicity studies on mice and rats [165-167]. In the Muta™mouse assay no mutagenic activity was observed although the liver weight of the treated mice increased indicating systemic effects in this organ [149,168]. Two studies are available on the Big Blue^® ^mouse, one gave negative [16] and the other weak positive results [169]. Taken together the results in transgenic mice are inconclusive which is in accord with the mouse bone marrow nucleus test (inconclusive results). Inconclusive (*in vitro*) or negative results were obtained in other studies on clastogenicity or other endpoints of genotoxicity (see Additional file 1).

Overall, there is no clear indication of gene or chromosome mutagenic activity in transgenic mouse assays or other genotoxic tests systems. However, genotoxic mechanisms in carcinogenicity cannot be excluded.

#### β-Propiolactone

This is an alkylating substance with predominantly local carcinogenic effects[175,176]. In the Muta™mouse assay [131] local effects in the stomach were observed in gavage studies plus systemic effects in the liver, but no mutagenic activity was seen in bone marrow of the Muta™mouse [131]. As expected, no increased incidence in micronuclei was detected in the bone marrow of treated mice [1,4] although clastogenic effects were observed in *in vitro *studies, in insects and plants (see Additional file 1) indicating chromosomal aberration after direct contact with this alkylating substance.

#### Trichloroethylene

This substance gave clearly positive results in different carcinogenicity studies on mice [186]. Although the target organs of carcinogenicity (including bone marrow) were investigated in the Muta™mouse assay, no mutagenic activity was noted [187]. The positive results in the mouse bone marrow micronucleus test are contradictory to other *in vivo *studies on clastogenicity. However, a further (simple) reason for the negative results in the Muta™mouse assay might be that the MTD was not reached [187]. Overall, further discussion on the mechanisms of carcinogenicity is necessary.

#### Tris(2,3-dibromopropyl)phosphate

This might be a further example for a substance where genotoxic effects are not related to the target organ bone marrow but induce systemic effects in others. The target organ in oral carcinogenicity studies on mice and rats was the kidney, in mice systemic carcinogenic effects were also seen in lung and liver [188-190]. In the Big Blue^® ^mouse assay, mutagenic activity was detected in the kidney in gavage studies[46,191]. The mouse bone marrow micronucleus assay (i.p. injection)[4] as well as cytogenetic studies on rats and mice were negative [188-190].

### Substance without data on carcinogenicity in mice and differing results in the micronucleus test and transgenic mouse assay

#### 4-Acetylaminofluorene

This substance showed mutagenic activity in the Muta™mouse assay [18] but inconclusive results in the mouse bone marrow micronucleus test [2]. No data on carcinogenicity are available on 4-acetylaminofluorene. However, data on two *in vitro *mammalian test systems indicated gene mutagenic activity [17] supporting results in the transgenic assay.

### Substances without carcinogenic effects in mice and differing results in the micronucleus test and transgenic mouse assay

#### Bromomethane

No increased tumour incidences were observed in mice as well as in re-evaluated studies on rats [58,59]. Negative results were obtained also in the examined organs including liver and bone marrow in the Muta™mouse assay. However, DNA methylation in the liver was observed in the same assay even at lower dose levels [60] indicating differences in the sensitivity of these two genotoxic endpoints. The mouse micronucleus test revealed chromosome mutagenic activity [61] although results of other *in vivo *tests are equivocal (see Additional file 1).

#### 2,6-Diaminotoluene

No carcinogenic effects were seen in a valid long-term study on mice and rats [85]. In the Big Blue^® ^mouse no mutagenic activity was induced [82,84]. However, only the liver was examined and the assays had some limitations (one dose tested, MTD possibly not reached). The mouse micronucleus test revealed chromosome aberrations [3,47] as well as the available *in vitro *studies but no clastogenic activity was detected in a cytogenetic study on rodents (see Additional file 1).

### Substances with carcinogenic effects in mice but nongenotoxic mechanisms are presumed

#### Chloroform (German MAK Classification 4 = substances with carcinogen effects where the genotoxic effects are absent or only play an insignificant role)

Liver tumours were induced in B6C3F1 mice [72], however no mutagenic effects were detected in the liver of Big Blue^® ^mice of the same strain in a valid assay [74]. Accordingly, no increased incidence in micronuclei were observed in the bone marrow of mice [1,47] and no clastogenicity in *in vitro *studies (see Additional file 1). In contrast, rats showed clastogenic activity in the kidney, but only at toxic dose levels [73].

#### Di-(2-ethylhexyl)phthalate (German MAK Classification 4)

This substance induced liver tumours in mice and rats [86]. No increased mutation frequency was seen in the liver of the Big Blue^® ^mouse [16] (limited validity, MTD presumably not reached) and no chromosome aberration in the mouse micronucleus test [4]. The majority of *in vitro *and *in vivo *tests revealed also negative results with di-(2-ethylhexyl)phthalate (see Additional file 1).

#### Tetrachloromethane (German MAK Classification 4)

An increased incidence of liver tumours were induced in mice and in rats [185]. However, no mutagenic activity in the liver was reported in a Muta™mouse assay although the organ weight increased in the same mice indicating some hepatocellular regeneration [168]. No chromosome aberration was induced in the mouse bone marrow micronucleus test [4]. Also other studies on this endpoint and the majority studies on other endpoint of genotoxicity revealed negative results (see Additional file 1).

### Discussion on the comparison of both assays

Most carcinogens in Additional file 1 are positive in transgenic mouse assays and in the mouse bone marrow micronucleus test although different endpoints are studied. This indicates coincidence in both test systems and/or effects of the test substance on different genotoxic endpoints.

However, there are several substances (see above for example ortho-anisidine) whose mutagenic (and carcinogenic) potential could not be demonstrated with the mouse bone marrow micronucleus test but with the transgenic mouse assay. This might be due to the fact that the micronucleus test is restricted to the bone marrow as target organ. In contrast, all organs can be examined in transgenic mouse assay for mutagenic activity without any restrictions.

A special subgroup of substances should also be mentioned: substances with predominantly local mutagenic/carcinogenic effects and less systemic direction of effects. For these substances the mouse bone marrow micronucleus test is an unsuitable test system.

Beside the restrictions on the target organ there is also given the possibility that a substance induces predominantly gene mutations and not or less chromosome aberrations at the same dose level. The example *N*-nitrosodi-*N*-propylamine has shown positive results in different target organs including the bone marrow using the transgenic mouse assay but negative results were shown in the mouse micronucleus test.

On the other hand there are also substances for which the transgenic mouse assay is an unsuitable test system. The examples methylmethanesulfonate and mitomycin C have shown that chromosome aberration and not gene mutation is the predominant endpoint at the corresponding dose level *in vivo*. These genotoxic effects are not easily detectable with the transgenic mouse assay which is restricted to the detection of small deletion and insertions in the DNA. However, the negative Muta™mouse assay on mitomycin C has some limitations in the validity of the test system. With increased dose levels (MTD reached) and/or repeated application also gene mutagenic effects might be detected in the transgenic mouse assay.

Interestingly, substances with carcinogenic effects induced by nongenotoxic mechanisms gave mainly correct negative results in both test systems although the protocols for the transgenic mouse assays were not optimised except for chloroform.

### Predictability of the transgenic animal assays and the mouse bone marrow micronucleus test for carcinogenicity

The sensitivity, specificity and predictive values to cancer for the Muta™mouse assay and the Big Blue^® ^mouse assay combined, and the mouse bone marrow micronucleus test are documented in Table 1. In the present study data on 38 substances were available concerning carcinogenicity in mice and mutagenic effects in transgenic mice as well as mutagenic effects in the mouse bone marrow micronucleus test (Additional file 1). The two substances with inconclusive results in the mouse bone marrow micronucleus assay (phenobarbital & acrylamide) were not included in the final calculation data and in the comparison of the micronucleus test versus the transgenic mouse assay.

**Table 1:** Characteristics of the Muta™mouse assay and the Big Blue^® ^mouse assay for predicting mouse carcinogenicity in comparison with the micronucleus test

Term#	Calculation for the mouse bone marrow micronucleus test	Calculation for Muta™mouse and/or Big Blue^® ^mouse combined *
Sensitivity	68% (23/34)	82% (28/34)
Specificity	0% (0/2)	100% (2/2)
Positive predictability	92% (23/25)	100% (28/28)
Negative predictability	0% (0/11)	25% (2/8)
Overall accuracy	64% (23/36)	83% (30/36)

**# **Sensitivity = % of carcinogens with a positive result in the specified test system (STS)

Specificity = % of noncarcinogens with a negative result in STS

Positive predictability = % of positive results in the STS that are carcinogen

Negative predictability = % of negative results in the STS that are noncarcinogens

Overall accuracy = % of chemicals tested where STS results agree with the carcinogenicity results

Carcinogens with genetoxic and nongenotoxic mechanisms were considered but not noncarcinogenic substances; only data on mice were used

Weak positive results in transgenic mouse assays judged as positive.

*: judged as positive in transgenic assays if positive in one of the two test systems

Although the data pool in this document is not sufficient for a comprehensive comparison (low number of examples, especially for specificity and negative predictability; limitations of most transgenic mouse assays with negative results) some differences were apparent between the two test systems.

The overall accuracy of the micronucleus test is lower than that of the transgenic mouse assays. This is mainly due to 11 negative results in the micronucleus test system (negative in the micronucleus test but positive in carcinogenicity studies) influencing the terms sensitivity and negative predictability. Three of these negative results in the micronucleus test are obtained with carcinogenic substances [chloroform, di-(2-ethylhexyl)phthalate, and tetrachloromethane] for which carcinogenic effects are considered to be of a nongenotoxic mechanism. However, chloroform, di-(2-ethylhexyl)phthalate and tetrachloromethane gave negative results in transgenic mice, so the comparison of both test system is not essentially affected and the evaluation „nongenotoxic“ supported. For the other 8 substances out of these 11 with false negative results in the micronucleus test these results are explainable (see above): Ortho-anisidine (mutagenic/carcinogenic effects are restricted to the bladder), IQ (bone marrow presumably not target organ of genotoxicity in mice and more gene mutagenic than clastogenic), asbestos (local genotoxic/carcinogenic effects in the lung), 2,4-diaminotoluene (target organ liver, presumably not bone marrow), N-nitrosodiethylamine (target organ liver, more gene mutagenic than clastogenic), N-nitroso-N-propylamine (presumably more gene mutagenic than clastogenic), beta-propiolactone (mainly local effects and less systemic effects [in bone marrow]), tris(2,3-dibromopropyl)phosphate (systemic effects not related to the bone marrow).

The term negative predictability is also low in the transgenic mouse assay due to false negative results on six carcinogenic substances; for three of them, hydrazine, mitomycin C, and tetrachloroethylene (detailed treatise in section „direct comparison“, see above) genotoxic mechanisms are presumed. For hydrazine (no repeated application) and tetrachloroethylene (MTD not reached) limitations on the experimentel design might be the reason for the negative results. Mitomycin C is clearly more clastogenic than gene mutagenic, and the transgenic mouse with lacI and lacZ is possibly an unsuitable test system. For 3 out of the 6 substances the carcinogenic effects in mice were attributed to nongenotoxic mechanisms: chloroform, di-(2-ethylhexyl)phthalate and tetrachloromethane (see also above), all gave negative results in transgenic mice.

Only two substances with negative results in long-term carcinogenicity studies are available in the data pool: bromomethane and 2,6-diaminotoluene. Both gave correct negative results in the transgenic mouse assay (although of limited validity) but false positive results in the micronucleus test (see term specificity).

Generally, the differences between the two test systems might be due to the fact that 1) unequal genotoxic endpoints are investigated (chromosome aberration in the micronucleus test versus gene mutation in the transgenic mouse assay), 2) organotrophy of genotoxic effects (especially bone marrow not target organ) might play an essential role and 3) transgenic rodent assay conditions in the different systems may not be optimal for mutation detection.

### Advantages and disadvantages of both test systems

A comparison of the transgenic mouse assays with the mouse bone marrow micronucleus test is limited by the fact that different genotoxic endpoints are studied in these two systems. In transgenic mouse assays, point mutations and small insertions and deletions are detected whereas in the mouse bone marrow assay, chromosome breakage leading to light microscopically visible micronuclei resulting from chromosome fragment or micronuclei originated from whole chromosomes are investigated.

#### Sensitivity of the test system

In comparison to other test systems in genotoxicity testing using endogenous target structures the spontaneous mutant frequency in the transgenic mouse assay is relatively high. This might be related to the fact that bacterial DNA is the target gene (high methylation rate) or the transgene is silent and no transcription related repair occurs like in endogenous genes which are more efficiently repaired [9]. In the mouse bone marrow micronucleus test the spontaneous rate of micronuclei is low ranging between 1–3 PCEs with micronuclei per 1000 PCEs. However, frequency of chromosome aberrations is not directly comparable with a gene mutantion frequency.

Comparing the target organs and cells at risk at the time of exposure, the mouse micronucleus test is restricted to one target organ, the bone marrow, especially to the erythroblasts. This limitation is not given in transgenic mouse assays: target cells are cells in all organs [195].

#### Considerations of animal welfare

Both test systems are similar in the number of animals used for a valid test. The minimal number of mice needed in the mouse bone marrow assay is 25 per gender (3 dose levels, vehicle control, positive control; 5 mice per group) using a treatment schedule with 2 or more applications at 24 h intervals and sampling 18–24 h following the final treatment. In the limit test (for a test substance of low toxicity) only one dose level of 2000 mg/kg bw is necessary (OECD guideline 474, [6]).

In transgenic mutation assays ca. 20 animals (3 dose groups and 1 concurrent vehicle control group in laboratories which already established this test system) are recommended per species and gender [196,197]. In terms of animal welfare, it is also desired to merge more than one in vivo genotoxicity assay such as transgenic mouse assay and micronucleus assay using the same animals for both assays.

#### Cost effectiveness

Due to the simplicity of the mouse bone marrow micronucleus assay and the use of systems for automated analysis, this test is less expensive than the transgenic mouse assay.

A comparison both test systems is presented in Table 2.

**Table 2:** Comparison of the mouse bone micronucleus assay with transgenic mouse models (Muta™mouse and the Big Blue^® ^assay)

	**Mouse bone marrow micronucleus test **[1,2]	**Transgenic mouse mutation assay **[9,195]
**Type of endpoint**	Detects light microscopically visible micronuclei resulting from whole chromosomes or chromosome fragments following chromosome breakage	Detects 1) gene mutation, 2) small deletions or insertions
**Regulatory use**	Widespread acceptance (OECD guideline established since 1983)	Not widely used by the industry in toxicological screening; OECD guideline in preparation
**Background mutation rate**	Spontaneous incidence of micronuclei is low (ca. 0.3%) and almost uniform	High spontaneous rate of mutations comparing with other mutation assays
**Negative predictivity**	low negative predictivity for cancer	Low negative predictivity for cancer
**Implementation**	Simplicity of the test system; easily recognised end-point	Higher Complexity of the test system (target cells in mice and expression of mutagenic effects in bacteria; vector system needed)
**Toxicokinetics and metabolism**	Restrictions in toxicokinetics: test substance or the toxic metabolites may not reach the bone marrow but other target organs	No restrictions after absorption and distribution of the test substance
**Target tissue**	Restricted to erythroblasts in the bone marrow	No tissue restriction; analysis of mutagenic potency in different organs; measurement of organotrophic effects
**Dependency of effects on application route**	Only systemic effects can be detected	Systemic as well as local mutagenic effects can be detected
**Number of animals**	5 animals per gender per dose recommended	5 animals per gender per dose recommended
**Restrictions on the used model**	Also some recommendations are given in OECD guideline 474, no limitation concerning species, strain, gender, age of animals, exposure duration	Limitations: Muta™mouse assay only 1 species & 1 strain; Big Blue^® ^2 species (mouse and rat) but 1 (rat) or 2 strains (mouse); no limitations on other parameters
**Costs**	Less expensive due to the simplicity of the test system	More expensive test system
**Molecular mechanism**	Mechanisms of the induction of micronuclei originating from chromosome fragments could not be resolved	Detection of the "molecular signature" of a particular mutagenic substance by DNA sequence analysis with standardised methods
**Parallel examination of different genetic endpoints**	Combination with other genotoxic endpoints is not recommended but possible if results of the micronucleus test are not influenced and vice versa	The transgenic mouse assay can be combined with other *in vivo *genotoxic endpoints in the same animal: micronuclei, chromosomal aberration, UDS, SCE
**Type of mutational target**	In situ end point	Target genes are integrated parts of foreign DNA and consequently no "normal" mutational target, no expression

UDS: unscheduled DNA synthesis; SCE: sister chromatid exchange.

## Conclusions

In a comparison of the tests available to genetic toxicologists, the results from studies on substances which had been tested in transgenic mutagenicity assays Big Blue^® ^mouse and the Muta™mouse were compared with those from the more traditional mouse bone marrow micronucleus test. The transgenic animal mutation assay, which is not yet used in toxicological screening, is not directly comparable with the micronucleus test, because different genetic endpoints are examined: chromosome aberration versus gene mutation. However, from the 39 substances, the majority gave the same positive or negative result in both test systems. The substances where differences occurred were discussed in more detail. The advantages and disadvantages of the transgenic Big Blue^® ^mouse and the Muta™Mouse transgenic model compared to the micronucleus test were discussed and both systems were found to have a place in mutagenicity testing and to supplement each other. The transgenic animal assay has, however, some distinct advantages over the micronucleus test in that it is not restricted to one target organ and detects systemic as well as local mutagenic effects.

## Authors' contributions

UW was the main author. The other authors were involved in the discussions, writing small parts of text and in final preparation of the manuscript.

## Supplementary Material

Additional File 1 Table: Results in the transgenic mouse assay versus mouse bone marrow micronucleus test Click here for file
