# Supplementary material for: Mutagenicity testing with transgenic mice. Part I: Comparison with the mouse bone marrow micronucleus test
Source: J Carcinog. 2005 Jan 17;4:3. doi: 10.1186/1477-3163-4-3 (PMC548135; doi:10.1186/1477-3163-4-3)
Supplement: Additional File 1 — Table: Results in the transgenic mouse assay versus mouse bone marrow micronucleus test [file 1477-3163-4-3-S1.doc]

Additional file 1: Results in the transgenic mouse assay versus mouse bone marrow micronucleus test

| Substance ^a)^ | **Results in carcinogenicity studies on mice [IARC evaluation]** | **Results in transgenic assays**  in all studied organs  (only in bone marrow) | | **Mouse bone marrow micronucleus test** | | | Further chromosome mutation assays | |
| --- | --- | --- | --- | --- | --- | --- | --- | --- |
|  |  | **Muta mouse** | **BB mouse** | **Results** | **Agreement with Muta mouse** | **Agreement with BB mouse** | ***in vitro*** | ***in vivo*** |
| 2-Acetylamino-fluorene^2,11-13^ | Positive  [no evaluation] | +^14^ (nd) | +^11,15,16^ (nd) | +^1^ | YES | YES | + | + (micronuclei, rat) |
| 4-Acetylamino-fluorene^17^ | no data  [no evaluation] | +^18^ (nd) | Nd | ±^2^ | inconclusive | na | nd | nd |
| Acrylamide^4,19,20^ | Positive  [2A] | +^21-23^ (+) | Nd | ±^4^ | inconclusive | na | ++ | + (micronuclei, mouse but not bone marrow) + (cytogenetic, mouse) + (dominant lethal, mouse & rat) + (heritable translocation, mouse) |
| Aflatoxin B1^24,25^ | Positive  [1] | nd | +^25-27$^ (nd) | +^2,28^ | na | YES | + | + (micronuclei, rat) + (cytogenetic, rat & mouse) ± (dominant lethal, mouse) + (dominant lethal, rat) |
| 4-Amino- biphenyl^1,29-31^ | Positive  [1] | +^32^ (+) | nd | +^4^ | YES | na | Nd | - (micronuclei, rat) |
| 2-Amino-3-methylimidazo(4,5-f)quinoline (IQ)^33^ | Positive  [2A] | +^34^ (nd) | nd | -^2,35^ | NO | na | ± | - (cytogenetic, mouse) + (cytogenetic, rat) |
| ortho-Anisidine^36^ | Positive  [2B] | nd | +^37^ (nd) | -^4,36^ | na | NO | + | - (micronuclei, rat) |
| Substance ^a)^ | **Results in carcinogenicity studies on mice [IARC evaluation]** | **Results in transgenic assays**  in all studied organs; (only in bone marrow) | | **Mouse bone marrow micronucleus test** | | | Further chromosome mutation assays | |
|  |  | **Muta mouse** | **BB mouse** | **Results** | **Agreement with Muta mouse** | **Agreement with BB mouse** | ***in vitro*** | ***in vivo*** |
| Asbestos crocidolite^38-40^ | Positive  [1] | nd | +^41^ (nd) | -^4^ | na | NO | ++ | nd |
| Benzene^2,42,43^ | Positive  [1] | nd | +^44-46^ (+) | +^2,47^ | na | YES | - | + (micronuclei, rat) + (cytogenetic, rat, mouse, human) - (cytogenetic, Drosophila) |
| Benzo(a)pyrene^48, 49,50^ | Positive  [2A] | +^51-54^ (+) | +^55-57^ (nd) | +^1,2,48^ | YES | YES | ++ | + (micronuclei, rat) + (cytogenetic, mouse & hamster) - (cytogenetic, rat)  + (dominant lethal, mouse) |
| Bromomethane^58,59, 61^ | Negative  [3] | -^60^ (-) | Nd | +^61^ | NO | na | + | + (micronuclei, rat) ± (micronuclei, human) - (cytogenetic, rat) - (dominant lethal, rat) |
| 1,3-Butadiene^62,66^ | Positive  [2A] | +^63^ (-) | +^63-65^ (+) | +^66,47^ | YES | YES | Nd | - (micronuclei, rat & human) + (cytogenetic, human, mouse) + (dominant lethal, mouse) - (dominant lethal, rat) + (heritable translocation, mouse) |
| Chlorambucil^2,4,67-71^ | Positive  [1] | +^21,22^ (+) | nd | +^2,4^ | YES | na | + | + (micronuclei, rat) + (cytogenetic, rat) ± (cytogenetic human) |
| Chloroform^72, 73^ | positive but nongenotoxic [2B] | Nd | -^74^ (nd) | -^1,47^ | na | YES | - | + (micronuclei, rat) + (cytogenetic, rat) ± (cytogenetic, mouse) |

| Substance ^a)^ | **Results in carcinogenicity studies on mice [IARC evaluation]** | **Results in transgenic assays**  in all studied organs; (only in bone marrow) | | **Mouse bone marrow micronucleus test** | | | Further chromosome mutation assays | |
| --- | --- | --- | --- | --- | --- | --- | --- | --- |
|  |  | **Muta mouse** | **BB mouse** | **Results** | **Agreement with Muta mouse** | **Agreement with BB mouse** | ***in vitro*** | ***in vivo*** |
| Cyclophos- phamide^1,75,76^ | Positive  [1] | +^21,22^ (+) | +^57,77-79^ () | +^1,2,47,80^ | YES | YES | ++ | + (micronuclei, rat) + (cytogenetic, rat, mouse, human) + (dominant lethal, mouse & rat) + (heritable translocation, Drosophila) |
| 2,4-Diamino- toluene^4,81^ | Positive [2B] | nd | +^82-84^ (nd) | -^4^ | na | NO | + | + (micronuclei, rat) - (dominant lethal, mouse) |
| 2,6-Diamino- toluene^85^ | negative(valid study) [no evaluation] | nd | -^82,84^ (nd) | +^3,47^ | na | NO | ++ | - (cytogenetic, rodents) |
| Di-(2-ethylhexyl) phthalate^86^ | positive but nongenotoxic [2B] | nd | -^16^ (nd) | -^4^ | na | YES | -- | - (cytogenetic, rat) + (cytogenetic, hamster) ± (dominant lethal, mouse) |
| 7,12-Dimethylbenz-[a]anthracene^2,87,88^ | Positive [no evaluation] | +^21,22,89-91^ (+) | +^92-95$^ (+) | +^2^ | YES | YES | + | + (micronuclei, rat) + (cytogenetic, rodent) |
| Ethylene oxide^1,96^ | Positive  [1] | nd | +^97,98^ (+) | +^1,99^ | na | YES | ++ | + (micronuclei, rat & human) + (cytogenetic, rat, mouse, human) + (dominant lethal, rat, mouse) + (heritable translocation, mouse, Drosophila) |
| Ethylmethane-sulfonate^1,100-103^ | Positive  [2B] | +^104-106^ (+) | nd | +^1^ | YES | na | ++ | + (micronuclei, rat) + (cytogenetic, mouse) + (dominant lethal, rat, mouse) + (heritable translocation, mouse, Drosophila) |

| Substance ^a)^ | **Results in carcinogenicity studies on mice [IARC evaluation]** | **Results in transgenic assays**  in all studied organs; (only in bone marrow) | | **Mouse bone marrow micronucleus test** | | | Further chromosome mutation assays | |
| --- | --- | --- | --- | --- | --- | --- | --- | --- |
|  |  | **Muta mouse** | **BB mouse** | **Results** | **Agreement with Muta mouse** | **Agreement with BB mouse** | ***in vitro*** | ***in vivo*** |
| N-Ethyl-N-nitrosourea^107,108^ | Positive  [2A] | +^22,63,105, 106,109,110^, (+) | +^111-117^ (nd) | +^2,118^ | YES | YES | ++ | + (cytogenetic, rat & mouse) + (heritable translocation, Drosophila) |
| Hydrazine &  hydrazine sulfate^119^ | Positive  [2B] | -^120^ (-) | nd | +^4,119^ | NO | na | ± | - (cytogenetic, mouse) + (cytogenetic, Drosophila) - (dominant lethal, mouse) |
| Methylmethane-sulfonate^2,121,122^ | Positive  [2B] | (+)^18,123-125^ (-) | -^111-113,126^ (nd) | +^1,127^ | YES | NO | ++ | + (micronuclei, rat) + (cytogenetic, mouse) + (dominant lethal, mouse) + (heritable translocation, mouse) |
| N-Methyl-N'-nitro-N-nitrosoguanidine (MNNG) ^2,128-130^ | Positive  [2A] | +^21,131,132^ (-) | nd | +^2^ | YES | na | ++ | + (micronuclei, rat) + (cytogenetic, mouse) - (dominant lethal, mouse) |
| N-Methyl-N-nitrosourea^133, 134^ | Positive  [2A] | +^135^ (nd) | +^136-138^ (nd) | +^1,4^ | YES | YES | ++ | + (cytogenetic, Drosophila) + (dominant lethal, mouse) + (heritable translocation, Drosophila) |
| Mitomycin C^1,139-141^ | Positive  [2B] | -^142^ (-) | Nd | +^1,47^ | NO | na | ++ | + (micronuclei, rat) + (cytogenetic, mouse) + (dominant lethal, rodents) + (heritable translocation, mouse) |
| 4-Nitroquinoline-1-oxide^28,135,143,144^ | Positive  [no evaluation] | +^135,145^ (+) | nd | +^1^ | YES | na | ++ | + (micronuclei, rat) |

| Substance ^a)^ | **Results in carcinogenicity studies on mice [IARC evaluation]** | **Results in transgenic assays**  in all studied organs; (only in bone marrow) | | **Mouse bone marrow micronucleus test** | | | Further chromosome mutation assays | |
| --- | --- | --- | --- | --- | --- | --- | --- | --- |
|  |  | **Muta mouse** | **BB mouse** | **Results** | **Agreement with Muta mouse** | **Agreement with BB mouse** | ***in vitro*** | ***in vivo*** |
| N-Nitrosodi-ethylamine^28,146-148^ | Positive  [2A] | +^104,106,149,150^ (-) | nd | -^1,4^ | NO | na | + | - (micronuclei, rat) - (dominant lethal, mouse) ± (heritable translocation, Drosophila) |
| N-Nitrosodi-methylamine^28,151,152^ | Positive  [2A] | +^153-155^ (nd) | +^37,126,137, 156-159^ (-) | +^2^ | YES | YES | + | ± (micronuclei, rat)- (cytogenetic, mammalia) ± (dominant lethal, rodents) + (heritable translocation, Drosophila) |
| N-Nitrosodi-N-propylamine^160-163^ | Positive  [2B] | +^164^ (+) | nd | -^4^ | NO | na | + | nd |
| Phenobarbital^165-167^ | Positive  [2B] | -^149,168^ (nd) | (+)^16,169^ (nd) | ±^2,4^ | inconclusive | inconclusive | ± | - (cytogenetic, mouse) |
| Procarbazine^170,171^ | Positive  [2A] | +^21,22,172, 173^ (+) | nd | +^1,174^ | YES | na | - | + (cytogenetic, mouse) ± (dominant lethal, mouse)  + (dominant lethal, Drosophila) - (heritable translocation, mouse & Drosophila) |
| ß-Propiolactone^175-178^ | Positive  [2B] | +^131^ (-) | nd | -^1,4^ | NO | na | + | + (cytogenetic, plant) + (heritable translocation, Drosophila) |
| Substance^a)^ | **Results in carcinogenicity studies on mice [IARC evaluation]** | **Results in transgenic assays**  in all studied organs  (only in bone marrow) | | **Mouse bone marrow micronucleus test** | | | Further chromosome mutation assays | |
|  |  | **Muta mouse** | **BB mouse** | **Results** | **Agreement with Muta mouse** | **Agreement with BB mouse** | ***in vitro*** | ***in vivo*** |
| Quinoline^179-182^ | Positive  [2A] | +^154,183^ (-) | nd | +^184^ | YES | na | + | - (micronuclei, rat) - (cytogenetic, mouse) + (cytogenetic, rat) |
| Tetrachloro-methane^185^ | Positive but nongenotoxic  [2B] | -^168^ (nd) | nd | -^4^ | YES | na | ± | - (cytogenetic, rat & mouse) |
| Trichloroethylene^186^ | Positive  [2A] | -^187^ (-) | nd | +^2,3^ | NO | na | ± | ± (micronuclei, rat) - (cytogenetic, rat & mouse) - (dominant lethal, mouse) |
| Tris(2,3-dibromo-propyl)phosphate^188-190^ | Positive  [2A] | nd | +^46,191^ (nd) | -^4,188-190^ | na | NO | ± | + (micronuclei, hamster) - (cytogenetic, rat & mouse) + (heritable translocation, Drosophila) |
| Urethane^2,192,193^ | Positive  [2B] | +^194^ (+) | +^137^ (nd) | +^2^ | YES | YES | + | + (micronuclei, rat) ± (cytogenetic, Drosophila) + (heritable translocation, Drosophila) |

## a): citations in this column related to data on carcinogenicity (2^nd^ column) and genotoxicity in vitro (8^th^ column) and in vivo (9^th^ column), data from secondary literature BB: Big Blue; -: negative study results; +: positive (for transgenic mouse assays: at least one examined organ shows an increased mutation frequency); ++ majority of results are positive concerning 2 or more endpoints in in vitro studies; (+): study result weakly positive; ±: inconclusive result; nd: not data available; na: not applicable because one test not done; $: positive also in BB rats
